# Development of a World Health Organization international survey assessing the lived experience of people affected by cancer: outcomes from pilot testing, user feedback, and survey revision

**DOI:** 10.1007/s00520-025-09372-2

**Published:** 2025-05-03

**Authors:** Clarissa E. Schilstra, Claire E. Wakefield, Jordana McLoone, Lori Wiener, Mark W. Donoghoe, Ruth I. Hoffman, Moses Echodu, Roberta Ortiz, André Ilbawi, Julie Cayrol

**Affiliations:** 1 Faculty of Medicine and Health, UNSW Sydney, Randwick, NSW Australia; 2 Behavioural Sciences Unit, Kids Cancer Centre, Level 1 South, Sydney Children’S Hospital, Randwick, NSW 2031 Australia; 3 National Cancer Institute, Bethesda, MD USA; 4 American Childhood Cancer Organization, Kensington, MD USA; 5 Uganda Child Cancer Foundation, Kampala, Uganda; 6 World Health Organization, Geneva, Switzerland; 7 Royal Children’S Hospital, Parkville, VIC Australia; 8 Murdoch Children’S Research Institute, Parkville, VIC Australia; 9 Department of Paediatrics, Faculty of Medicine, The University of Melbourne, Parkville, Australia; 10 Division of Quality of Life and Pediatric Palliative Care, Department of Pediatrics, Stanford University and Stanford Medicine Children’s Health, Palo Alto, CA USA

**Keywords:** Lived experience, Cancer, Global research survey

## Abstract

**Purpose:**

Assessing differences between lived experiences of people affected by cancer internationally facilitates direction of international health policies and standards. The study piloted, on behalf of the World Health Organization (WHO), a global survey assessing the lived experience of people affected by cancer. We aimed to determine (1) the acceptability of the survey and (2) the survey’s capacity to capture a globally representative sample of people diagnosed with cancer.

**Methods:**

The cross-sectional survey went through two pilot rounds. We (1) solicited feedback from international cancer organisations through a feedback form, and (2) launched a global online survey, requesting open-ended feedback on the survey format/content from people diagnosed with cancer, their family members/caregivers, and bereaved family members.

**Results:**

*Round one*: 23 stakeholders found the survey acceptable in length/content. Minor suggestions were to improve readability/applicability across healthcare settings. *Round two*: 505 individuals participated: 177 (35%) provided feedback on the study design (e.g. to include people currently being treated for cancer, and siblings) or survey (e.g. assessing impacts of multiple cancers). Participants seemed to value the opportunity to share their experiences: “Thanks…felt good to answer as if someone was listening.” Compared with global statistics, our sample of people diagnosed with cancer (*N* = 240) included significantly more females (*p* < 0.001) and individuals from high-income countries (*p* < 0.001).

**Conclusion:**

Participant feedback informed important changes to the survey design and content. Our findings highlight that engaging with people with lived experience is a critical first step to develop such a global survey, optimise participation, and amplify individuals’ voices.

## Introduction

The global incidence of cancer now exceeds 19 million people per year [1–3]. While significant treatment advances in the last two decades have contributed to rising survival rates (now over 70% in many high-income countries, HIC), cancer also accounts for nearly 10 million deaths globally each year [1, 3]. Behind these numbers are individuals, families, and communities experiencing cancer, caring for a loved one with cancer, or grieving the loss of a loved one to cancer. Most research focused on understanding the lived experience of people affected by cancer has been limited to studies conducted in individual HICs, with questions focusing on country-specific healthcare experiences, especially around clinical processes, of people diagnosed with cancer themselves. Although these population-level studies provided novel and impactful insights into the determinants of cancer care experiences in these HICs, such studies have, to-date, lacked representation of the quality of life outcomes or lived experiences of people affected by cancer, including family members of those diagnosed, across low-, middle-, and high-income countries [4–7]. We therefore lack a global perspective on people’s lived experience, particularly in the areas of social, emotional, and financial well-being, which are negatively impacted for people with cancer and their family members/caregivers, and associated with unmet need [4–6, 8, 9].

The ‘lived experience of people affected by cancer’ is defined here as the experience of living with, or having lived with cancer, including but not limited to the experience of receiving treatment for cancer. It also includes supporting a loved one through cancer, both during cancer treatment and in the long-term, after treatment has finished or after a loved one has died. Conducting a global study on the lived experience of people impacted by cancer provides an invaluable opportunity to assess differences between lived experiences in high- and low-income settings, with potential to direct health policies relating to cancer care globally, identify opportunities for improvement in international standards for cancer care, and improve health equity among those affected by cancer. These are primary priorities for the World Health Organization (WHO) Global Action Plan for the Prevention and Control of Non-communicable Diseases 2013–2020 [10] and the 2030 United Nations Sustainable Development Goals [11].

The World Health Organization (WHO), alongside key partners, therefore commenced a program of work aiming to better understand the immediate and long-term social, emotional, and financial impact of cancer on people diagnosed with cancer at any age, their family members and caregivers, and the family members of those who have died from cancer, in both HICs and low-middle income countries (LMICs) [12]. The program is part of a WHO campaign for the Meaningful Engagement of People Living with Non-communicable Diseases [13]. The first step in this program of work was the development of a global research survey to understand the lived experiences of people affected by cancer, with particular focus on their psychosocial and financial well-being.

Increasing evidence suggests that engaging with people with lived experience of illness to understand their perspectives on the feasibility and acceptability of a research survey is critical to ensuring the survey’s relevance, readability, and understandability for future participants [14]. Therefore, prior to full launch of the WHO survey, we piloted the survey in two stages, to seek feedback from stakeholders and people with lived experience of cancer themselves, or as family members of people diagnosed with cancer. The primary aim of this pilot was to assess the acceptability of the survey, from the perspectives of people affected by cancer around the world (Aim 1), as well as to achieve broad representation of people diagnosed with cancer across sex, cancer type, and country income level (high, middle, or low, according to World Bank country income classifications) (Aim 2) [15].

## Methods

### Design

**Fig. 1:**
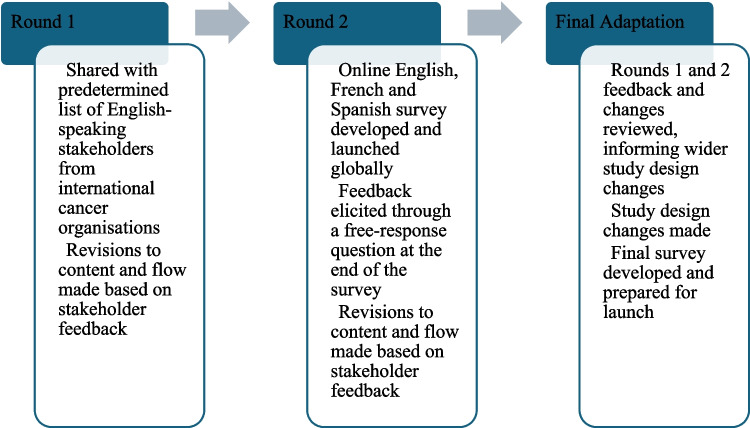
Pilot rounds

This global, cross-sectional, survey was developed in collaboration between the WHO and a steering committee comprising three cancer physicians, three psycho-oncology researchers (one of whom had a lived experience of a childhood cancer diagnosis and two of whom had lived experience of caring for a family member with cancer), one statistician, one parent of a childhood cancer survivor, and one childhood cancer survivor. The measures included in the survey are described in Table 1 [12]. The questionnaire domains were chosen using the WHO Quality of Life Framework [16], together with a literature review and steering committee consensus. We also consulted with WHO leaders from the Global Initiative for Childhood Cancer to ensure alignment of the survey with the campaign for the Meaningful Engagement of People Living with Non-communicable Diseases [13]. The survey includes validated measures and open-ended questions assessing individuals’ clinical and demographic characteristics, as well as their social, emotional, and financial well-being. The survey was developed in English and translated into French and Spanish by the research team. The survey was approved by the WHO Ethics Review Committee and deemed exempt from formal review given its low-risk nature. The survey went through three phases of change: pilot round one, pilot round two, and final adaptation based on pilot feedback (Fig. 1).

**Table 1:** Survey domains and measures

Domain	Measure and response option	All people affected by cancer	Survivors	Family members of people affected by cancer	Bereaved family members
Introductory questions	Forced choice options. Items purposely developed	**X**	**X**	**X**	**X**
Demographics	Mixture of forced choice options with options to add free text (e.g. ‘other’), depending on the question. Items either purposely developed or based on Childhood Cancer Survivor Study or the FOCUS study	**X**	**X**	**About self**	**About self**
Clinical information about person with cancer	Mixture of forced choice options with options to add free text (e.g. ‘other’). Items based on the FOCUS study	**X**	**X**	**About their family member (excluding types of treatments received)**	**About their family member (excluding types of treatment received)**
Survivorship care experiences	Items based on the FOCUS study	**X**	**X**	**-**	**-**
Fertility experiences of person with cancer	Mixture of forced choice options with options to add free text (e.g. ‘other’). Items purposely developed	**X**		**-**	**-**
Perceived impact of cancer	Items based on the FOCUS study	**X**	**X**	**-**	**-**
Impact on marriage/relationship (if applicable)	Purposely developed	**X**	**X**	**X**	**X**
Education/work problems	Adapted from Long-Term Follow-Up Study questionnaire and purposely developed	**-**	**X**	**X**	**X**
Caregiver burden	Family Appraisal of Caregiving Questionnaire for Palliative Care (FACQ-PC) by Cooper et al. 2006, and an additional item purposely developed			**Self**	**Self**
Prolonged grief	Prolonged Grief Scale by Prigerson et al. 2021	**-**		**-**	**Self**
Impact of cancer on siblings	Sibling Cancer Needs Inventory (Patterson et al. 2014) and purpose-designed questions (11 items)	**-**		**Siblings only**	**-**
Health-related quality of life	PROMIS Global	**X**		**Self**	**Self**
Health behaviours	Purposely developed	**X**	**X**	**Self**	**Self**
Emotional problems	Purposely developed	**X**	**X**	**Self**	**Self**
Mental health	Purposely developed	**X**	**X**	**Self**	**Self**
	Items based on the FOCUS study	**X**	**X**	**Self**	**Self**
	PROMIS Anxiety short form	**X**	**X**	**Self**	**Self**
	PROMIS Depression short form	**X**	**X**	**Self**	**Self**
Financial well-being	Purposely developed	**X**	**X**	**Self**	**Self**
	COST measure developed by De Souza et al. 2017	**-**	**X**	**Self**	**Self**
Support service use	Based on a National Cancer Institute Survey	**X**	**X**	**Self**	**Self**
End of life care experience	Purposely developed	**-**	**-**	**-**	**Self and family member**
Post-traumatic growth	Purposely developed	**X**		**Self**	**Self**
Missed topics	Purposely developed	**X**		**Self**	**Self**

### Aim 1: assess the acceptability of the survey, from the perspectives of people affected by cancer around the world

#### Pilot round one

##### Eligibility criteria and recruitment

The steering committee developed a list of stakeholders representing people affected by cancer from organisations around the world, aiming to obtain feedback from a range of countries, roles (survivors and family members), and professions (researchers, medical and allied health clinicians). Stakeholders were required to be over the age of 18 and were emailed an invitation to provide feedback on the survey from a member of the steering committee who was familiar with or already connected to them professionally. Following consent, stakeholders were emailed a Microsoft Word copy of the survey. All invited stakeholders were English speakers, although not all from English-speaking countries, who reviewed the English version of the survey.

##### Measures

We requested feedback on the feasibility and acceptability of the survey through a brief set of questions. Feasibility questions asked participants to rate on a scale of 1–5 (‘very easy’ to ‘very difficult’) how difficult it would be to complete the survey. Further questions asked about the acceptability of the survey length, suggestions for improving the survey (e.g. addition of missing topics), and research questions of interest for stakeholders (e.g. understanding differences in fertility information provision and care internationally).

##### Analysis

All feedback was analysed using NVivo software [17]. Participants’ responses were grouped using inductive content analysis [18] that formed categories based on (1) the feasibility question scale and (2) suggestions to improve the acceptability of the survey. Inductive content analysis involves using pre-determined categories to code and organise the data [18], which facilitates understanding of both the types of feedback and the quantity of each type of feedback.

#### Pilot round two

##### Design

Based on pilot round one feedback, revisions were made to the survey. An online version of the survey was built using LimeSurvey software [19] and hosted online by WHO. The survey remained live from October 18, 2022, to September 30, 2023.

##### Eligibility criteria and recruitment

Participants were recruited internationally through a combination of convenience and snowball sampling, via WHO networks, project stakeholders, and organisations who participated in pilot round one. We disseminated the survey in English, French, and Spanish, via press release by WHO Headquarters, to all member countries and regions [20]. Eligible participants were also recruited by (1) direct email invitation sent from the steering committee, (2) email invitation through a WHO network, project stakeholder, or partner organisation, or (3) accessing the survey directly through the WHO website.

Eligible participants included those who were over the age of 18 and (1) had been diagnosed with cancer (in childhood or adulthood) and finished cancer treatment, or (2) were a family member (spouse, parent, child, sibling, or other primary caregiver) of a person diagnosed with cancer and who had finished cancer treatment, or (3) were a family member (spouse, parent, child, sibling, or other primary caregiver) of a person who had died from cancer. Children or siblings under the age of 18 and people currently receiving cancer treatment (e.g. chemotherapy, radiation therapy, stem cell therapy) were excluded from this pilot.

##### Measures

To assess the acceptability of the survey from participants’ perspectives, we obtained feedback through a free-text box in which participants could provide comments and suggestions, which asked: “You have now come to the end of the survey. We would appreciate learning about any concerns (e.g. question wording) that you may have, or points of clarification you would like to make about your responses to specific items (e.g. if the response options didn’t seem to “fit” your experience). Also, please let us know if there were topics you thought should be covered but were not. Any other feedback is welcome, as well.”

##### Qualitative analysis

Qualitative data obtained from the open-ended questions was analysed using deductive content analysis, which involves forming categories directly out of the data, in NVivo software [17, 18]. CS and JC each independently coded the feedback to derive categories, then discussed any discrepancies to define a final list of categories, and JC re-coded the data, where needed, into the final list of categories. Categories were included if two or more participants provided the same kind of feedback.

##### Revision

Revision of the survey according to pilot feedback involved tabulating all qualitative feedback from the pilot rounds and making changes accordingly.

### Aim 2: achieve broad representation of people diagnosed with cancer across sex, cancer type, and country income level (high, middle, or low, according to World Bank country income classifications)

Descriptive statistics were computed to characterise participant demographics, including age, sex, and residential country. Similarity of the sample relative to international cancer normative data was assessed by comparing the data on cancer survivors from our sample to data from the 2022 Global Cancer Statistics [3], across sex, country income level, continent, and cancer type (haematological, solid tumour, or central nervous system tumour) using chi-squared goodness-of-fit tests.

## Results

### Aim 1: acceptability of the survey from the perspective of people affected by cancer internationally

#### Pilot round one

An email requesting feedback on the feasibility and acceptability of the survey was sent to 33 stakeholders who spoke English and could review the English version of the survey. A total of 23 stakeholders from across HIC and LMIC completed the survey: including 3 from low-income, 7 from middle-income, and 13 from high-income countries. Stakeholders included 9 cancer survivors, 9 clinicians and/or researchers (including 5 psychologists, 2 public health researchers, and 1 nurse), 3 cancer advocates (health administration or non-profit organisation professionals) without direct relation to someone who had cancer, 2 caregivers of cancer survivors, and 1 cancer survivor who is also a bereaved caregiver of someone who died from cancer. In terms of representation across WHO regions, 3 stakeholders were from the African Region, 12 from the Americas, 4 from Europe, 1 from the Eastern Mediterranean, and 3 from the Western Pacific. No stakeholders were from the South-East Asian Region.

Feedback was generally positive, with more than half of stakeholders (*n* = 14, 61%) reporting the survey length was appropriate and most (*n* = 19, 83%) reporting there were no topics missing. However, 11 (48%) reported it would be “somewhat difficult” or “extremely difficult” to complete the survey, while 8 (35%) reported it would be “somewhat easy” or “neither easy nor difficult”. All 23 stakeholders provided one or more suggestions for improving the survey. These included improving the readability and international applicability of the survey questions, recommending simplification of wording and removal of any medical jargon, and removal of questions about survivorship care plans as models of survivorship care are too variable internationally.

##### Survey revision: pilot round 1

Based on the feedback from pilot round 1, the survey was adapted, with some items removed, others added, and some questions being reformulated. Specific changes to survey items as well as the addition of items in response to the feedback described are summarised in Table 2.

**Table 2:** Pilot round 1 feedback

Category	Count	Example	Response to feedback
Pilot round one			
Simplify survey language, add more options for “I don’t know” in case participants are not sure how to answer questions, and be clearer about the time actually required to complete the survey	23	“Overall, this is beautiful and thoughtful work that will be a wonderful contribution on so many levels. I have added specific comments throughout but a few issues to consider overall: (1) simplifying language to align with health literacy recommendations. This includes choosing simpler words and reducing complexity of sentence structures (during introductory instruction sections, for example); (2) adding more options for “I don’t know” or “not sure”, as many respondents may not know dates or even types of cancers, etc.; just a little more allowance for uncertainty; (3) reconsidering survey time—I suspect it may take respondents longer to respond to all the questions than the proposed times.” **“**Possibly to minimise the burden (in terms of length) reflecting on the inclusion of some items which might overlap e.g. lots of measures cover anxiety”	The survey text was simplified where possible (unable to change text in validated measures), “Not sure” and “I don’t know” options added to almost every question, and redundant questions/measures were removed

Items that were removed to improve the flow, reduce the length of the survey, and ensure applicability to all healthcare contexts included: items on the perceived quality of care and support received during and after treatment, specific items on family members’ cancer treatment, and some caregiving questions. Questions relating to survivorship care and feelings experienced by survivors in this regard as well as some of the perceived professional qualities in follow-up care were also removed, given the variability of access to survivorship care globally. Changes were made to language to simplify or define medical terminology (e.g.: “metastatic”, “hereditary cancer syndrome”, definitions of certain medical specialists).

#### Pilot round two

We received responses from 607 individuals, but excluded responses that were entirely incomplete (i.e. blank, *n* = 100) or appeared suspicious with odd language/statements (i.e. incoherent text, content not related to cancer or the survey; *n* = 2). This resulted in a total of 505 responses included for analysis. Given the anonymous nature of the survey, we were not able to verify individuals’ identities or responses.

### Participant characteristics

Of the 505 included responses, participants were primarily female (419/505, 83%), identified as being a cancer survivor (301/505, 60%), and spoke English as a first language (379/505, 75%) (Table 2). Of the 301 who identified as a cancer survivor, 240 chose to respond to the survey as a cancer survivor and 61 chose to respond to the survey as a family member of someone who had cancer. Most participants were from high-income, English-speaking countries (368/505, 73%) (Table 3).

**Table 3:** Participant characteristics (*n* = 505)

Age	*Range* = 18–88, *M* = 48 (SD = 13.41)
Sex	Female: 419/505 (83%) Male: 83/505 (16%) Missing: 3/505 (1%)
First language of participants	English: 379/505 (75%) French: 29/505 (6%) Spanish: 17/505 (3%) Other: 80/505 (16%)
Cancer experience	Identifies as a survivor and family member of someone who had cancer: 179/505 (35%) Identifies as a cancer survivor only: 122/505 (24%) Identifies as family member only: 204/505 (40%) *Chose to respond as:* Cancer survivor: 240 (47%) Family member: 265 (52%)
Cancer survivors’ diagnoses	Solid tumour: 166/240 (69%) Central nervous system tumour: 5/240 (2%) Haematological malignancy: 45/240 (19%) Other: 9/240 (4%) Missing: 15/240 (6%)
Country income level	High income/English-speaking: 368/505 (73%) High income/non-English speaking: 78/505 (15%) Middle income (includes upper-middle and lower-middle income): 52/505 (10%) Low income: 0 (0%) Missing: 7 (1%)
WHO region	African Region: 7 (1%) The Americas: 292 (58%) European Region: 9 (19%) Eastern Mediterranean Region: 8 (2%) Western Pacific Region: 68 (13%) South-East Asian Region: 26 (5%) Missing: 7 (1%)

### Free-text feedback on the survey

Feedback was provided by 177/505 (35%) participants, while 333/505 (66%) left the feedback field blank or indicated N/A in the feedback field, and 5/505 (0.9%) indicated that they were happy with the survey as it was. Categories derived from review of the feedback are described in Table 4. Of the 177 participants who entered a response, 112 (112/177, 63%) provided more general comments relating to their personal lived experiences of cancer not applicable to the survey. Sixty-five participants (65/177; 38%) provided feedback on the survey itself, which we were able to use to directly inform improvements to the survey content and flow.

**Table 4:** Feedback from participants

Category	Count	Example	Response to feedback
Pilot round two			
Provided responses including “N/A”, “none”, or “not applicable”	20		
Provided positive feedback and were happy with the survey	21	“Thanks for the questions. Felt good to answer as if someone was listening.” “I think the survey is very well designed covering every aspect of life. Kudos to the team working for the betterment of the cancer survivor.”	
Left the feedback field blank	287		
Survey did not include people currently receiving cancer treatment	5	“I hope that WHO might consider doing a separate survey to understand the needs of people with metastatic or advanced cancer, especially those such as breast, prostate, melanoma and colorectal who are now living much longer with what remains an incurable cancer.”	People living with cancer included and added to eligibility list at the beginning of the survey
Discomfort with positively framed questions	9	“I really oppose the use of the word positive. There is NOTHING positive, there’s no silver lining to cancer let alone pediatric cancer. This isn’t a learning opportunity.” “The questions about what “positive” things came out of this whole experience feel very wrong and are almost angering to me.”	Perceived positive language rephrased to be more neutral. e.g.: “Please tell us about any positive impact” Care-giving question “How happy are you to do these things?” removed
Survey did not account for all potential mental health impacts	8	“An additional focus on PTSD would have been nice.” “I would have liked to see more of an emphasis on trauma.”	Open-ended item relating to mental health impacts added
Initial survey information did not state expected completion time	2	“An approximation of time to complete the survey—I was expecting 5–10 min, not 30 min.” “It’s very long and I hope there something that comes out this for everyone’s benefit.”	Information paragraph at the start of the survey added to inform of approximate completion time Development of a participant information statement to be translated and distributed to organisations, partners and participants, as part of dissemination plan
Survey did not account for changes in responses over time	9	“I based my answers on my life at or near the point when my daughter had cancer. She completed her treatment 15 years ago, so some things have changed.” “Some of my responses would have been different 10 or 15 years ago.”	Details added in question prompt to guide participants on the timing of the response to each question (e.g.: How would you rate your fatigue **at this time**?”)
Survey did not account for death of family member/loved one due to cancer disease versus cancer treatment	2	“This questionnaire was not designed to include those who died as a result of cancer treatment, such as my son, but I completed it anyway.” “There were no questions that addressed situations where the cause of death was related to the cancer treatment instead of cancer itself. My daughter died because of chemotherapy toxicity.”	Item added on the reason/cause of death
Survey did not account for different responses based on different or multiple cancer experiences	10	“I answered this question on behalf of my son, but in my experience caring for my husband I might answer some of the questions differently.” “Perhaps include an option to include extra diagnosed cancers. I have had three diagnosed cancers.”	Items added to clarify number of different cancer experiences. (e.g.: Please list all the cancer diagnoses your family member has received. What year was their latest cancer diagnosis?”)
Survey did not account for impact of cancer on employment	7	“My employment struggles related to returning to work were not included.” “The impact of breast cancer on work was the toughest experience!”	Items regarding employment and additional financial/employment support added. (e.g.: “How supported by your employers did you feel after your cancer treatment?”)
Survey did not account for items not being applicable to childhood cancer survivors	5	“I was 4 so the relationship questions and financial questions are not applicable.” “I was a child when my brother had cancer so question about how his cancer impacted my financial situation or my relationship with my partner or caring for him were not relevant.”	Details added in question prompt to guide participants (e.g.: “If you had cancer as a child, did your parents/guardians received information on fertility pertaining to you?”)
Survey did not account for recent versus long ago loss of family member	2	“Should be different for parents with recent loss & those who lost a child long ago.” “My answers reflect the fact that my child died in 2001. If you had asked me these questions now my answers would be very different.”	Addressed in the same way as the above category on changes in responses over time
Survey did not account for specific sibling experiences from parents’ perspectives	6	“I’d like to speak to the impact on her sibling.” “My child who is living has suffered from extreme anxiety, ptsd, and lots of issues as a result of the death of her younger sister. She was 17 when her only sibling passed away. I knew it would be difficult for all of us, but I wasn’t prepared for the amount of trauma that she had endured.”	Open-ended item added relating to the impact on siblings from parent’s perspectives
Survey did not account for variability or change to marriage/relationships	*11*	“I was already divorced when diagnosed at 46 but was dating a psychiatrist for six months.” “I was married to my son’s father during my son’s cancer treatment. However, I have since divorced and remarried. So, I am married now, but the cancer diagnosis had no impact on this marriage. The survey did not allow for that sort of situation.”	Changes made according to feedback to allow different options to marriage/relationship questions
Technical feedback (i.e. survey logic, response option order)	30	“The sliding scale didn’t work well on my phone. I put 10% for percentage of income spent, which may not be accurate because of this.” “I am currently unable to work because I was disabled in an accident. There seemed to be a glitch in that portion of the survey with the “Other” box saying it hadn’t been properly filled, so I think I eventually selected that I was a full-time homemaker so I could get on with the survey.”	Technical changes made, testing of survey by additional team members, and fixing of specific logic errors
Specific lived experiences not related to the survey content or design	70	“Getting back to normal life after cancer is hard. You look ok but inside you do still worry about recurrence. Also, I don’t want to be defined by my cancer, but I don’t want people to dismiss it either as I still have side effects and days when I’m tired and lots of menopausal symptoms. People in work think I should be fine now and able to work as I did previously but that is not the case” “Having an 8-month-old diagnosed with cancer was like a bomb going off in my life. It completely destroyed so many aspects of my life that I’m still picking up the pieces from 1.5 years later.”	These responses did not suggest revisions to the survey or topics that were missing, but rather highlighted how different lived experiences can look for people affected by cancer, and how much participants were willing to share in the hopes it could improve the experiences of others

Most participants in pilot round 2 did not provide feedback, or indicated they were happy with the survey as it was (Table 4), sharing statements such as “Thanks for the questions. Felt good to answer as if someone was listening.” Constructive feedback in pilot round 2 centred around aspects of lived experiences that the survey did not account for, such as impacts of cancer on employment, responses based on different or multiple cancer experiences, impact of childhood cancer on siblings, and experiences of people living with metastatic or advanced cancer where treatment status as identification as a “survivor” may be less clear. For example, a parent of a childhood cancer survivor shared the challenging impact of the death of their child with cancer, on their sibling: “My child who is living has suffered from extreme anxiety, PTSD, and lots of issues as a result of the death of her younger sister…I wasn’t prepared for the amount of trauma that she had endured.” And a person living with metastatic cancer shared: “I hope that WHO might consider including the needs of people with metastatic or advanced cancer…who are now living much longer with what remains an incurable cancer.” Other perspectives participants felt were missing included recent versus long ago loss of a family member, change in marital relationships over time, impact of other chronic illnesses on the cancer experience, impact of mental health conditions on people’s long-term lived experience, and how responses to certain questions may change over time. Technical feedback was provided by a subset of participants as well (30/505, 6%), including requests for improvement to response layouts and survey flow (grouping of similar types of questions).

While we collated and considered all suggested additional topic areas, we did not include some topics in the final version of the survey because they were not aligned with the study focus, or they would significantly increase the survey length. Additionally, following pilot round two, the research team was contacted directly by more than 10 cancer organisations and partners, requesting that people currently living with cancer and their family members be included, to capture their voices and experiences. Together, these challenges required revision to our study design across recruitment approaches, stakeholder engagement, and accessibility of the survey (Table 5).

**Table 5:** Changes to survey design based on pilot rounds

Challenge	Changes
Inclusion of people currently living with cancer	• Inclusion of people currently living with cancer in the survey introduction/inclusion criteria • Rephrasing of questions to be inclusive of people currently living with cancer and those that are in remission/cured/having no evidence of disease
Maintenance of communication with stakeholder organisations across the study recruitment period	• Development of a dissemination committee comprised of key stakeholders from LMICs that meets monthly through the recruitment period • Identifying additional individuals who can serve as study champions in LMICs to further support recruitment by disseminating advertising materials • Development of a communication package with invitations, flyers, email templates, and social media tools to support organisations in disseminating the survey
Accessibility of the survey	• Translation into 4 additional languages beyond English, Spanish, and French, including Arabic, Chinese, Russian, and Brazilian Portuguese • Development of brief versions of the survey that will enable printing of surveys by partner organisations in areas where internet access is limited, and an ethics-approved anonymous survey return system supported by partner organisations • Sending multiple reminders to organisations rather than one single press release/invitation to disseminate the survey

### Aim 2: achieve broad representation of people diagnosed with cancer across sex, cancer type, and country income level (high, middle, or low, according to World Bank country income classifications)

Compared with international normative data on people with cancer [3], our sample of 240 cancer survivors included significantly more females (83% in our sample compared to 48% internationally, *X*^2^ (1) = 71.3, *p* < 0.001), a higher proportion of individuals from high-income countries (88% vs. 21%, *X*^2^ (1) = 47.1, *p* < 0.001), and a higher proportion of individuals from the Americas (40% vs. 13%, *X*^2^ (1) = 151, *p* < 0.001). Our sample included significantly fewer survivors with a solid tumour diagnosis (69% vs. 80%, *X*^2^ (1) = 17.6, *p* < 0.001) and more survivors with a haematological diagnosis (leukaemia, lymphoma, or myeloma) (18% vs. 6.1%, *X*^2^ (1) = 64, *p* < 0.001). However, the proportion of CNS tumour diagnosis (1.2% vs. 1.6%, *X*^2^ (1) = 2.28, *p* = 0.13) in our sample did not significantly differ from international data.

## Discussion

This novel, global study was initially piloted with 23 stakeholders, the first version of the survey yielded generally positive feedback, with most participants (> 60%) finding the length and content of the survey acceptable. Their suggestions for improvement centred around simplifying the wording of the survey to limit medical jargon and improve its readability. Following these revisions, the second pilot yielded similarly positive results with most participants (57%) leaving the feedback field blank or sharing positive feedback indicating that they were satisfied with the survey length, content, and flow (8%). Constructive feedback was provided by a subset of participants (13%) through which they requested inclusion of additional aspects of lived experience (e.g. mental health, employment, other illnesses, multiple cancers, death due to cancer disease versus cancer treatment), as well as technical improvements to response layouts and survey flow. Participant demographics and feedback informed further study design changes, such as inclusion of people currently being treated for cancer and targeted approaches to more effectively recruit from LMIC. These adjustments aim to ensure that future participants, regardless of their location or background, feel seen and understood.

### Limitations

The representativeness of the sample of participants who completed the survey was limited. Input from males and individuals from LMICs was limited. The voices heard were predominantly those of females from high-income, English-speaking countries, which highlighted a gap we are keen to bridge in future survey efforts. These limitations are likely due to the limited availability of the survey in English, French, and Spanish, and the limited nature of dissemination pathways for the survey. Dissemination of the survey was conducted through WHO social media channels and the WHO website, as well as convenience sampling through a limited number of cancer organisations known well by the research team. No targeted or tailored dissemination of the survey in LMIC was conducted. This likely skewed our findings, contributing to the significant differences we found between people diagnosed with cancer in our study population and global statistics. Based on our snowball/convenience sampling approach, there is also likely response bias, and we cannot know who chose not to review/participate in the survey. This issue was likely exacerbated by the languages in which it was provided, as evidenced by lack of participation from people in LMICs and the predominance of participants from English-speaking backgrounds and countries. This limited the generalisability of our results and highlighted a need for us to translate the survey across additional languages, beyond English, French, and Spanish. Furthermore, these results prompted us to identify dissemination pathways in LMICs, to ensure the survey reaches these individuals whose voices are typically least heard in cancer research.

Consistent with existing research on the value of engaging people with lived experience in research study design [14], our engagement with people diagnosed with cancer, or their family members, has proven to be extremely valuable. Lived experience feedback has provided us direction to improve the survey’s acceptability and capture a wider range of experiences, across more sex/gender identities, from a more diverse set of countries and cultures, and in more languages. This pilot was a critical first step in understanding how we can optimise the launch of and participation in this kind of global study in the future.

### Future directions

By revising the survey, translating it into additional languages, and creating a dissemination plan that involves a communication package and dissemination committee of stakeholders from across high-, middle-, and low-income countries, we aim to tailor and target dissemination of the survey, and to further improve the reach and acceptability of this global survey to assess the lived experience of people affected by cancer. Use of probability-based sampling, although more time and resource intensive, along with dissemination committee, may support improvement of the representativeness of the sample collected through this kind of global study in the future.

## Data Availability

No datasets were generated or analysed during the current study.
